# Within-Flock Population Dynamics of *Dichelobacter nodosus*

**DOI:** 10.3389/fvets.2017.00058

**Published:** 2017-04-24

**Authors:** Edward M. Smith, Andrew Gilbert, Claire L. Russell, Kevin J. Purdy, Graham F. Medley, Mohd Muzafar, Rose Grogono-Thomas, Laura E. Green

**Affiliations:** ^1^School of Life Sciences, University of Warwick, Coventry, UK; ^2^School of Veterinary Sciences, University of Bristol, Langford, UK

**Keywords:** footrot, *Dichelobacter nodosus*, strain variation, MLVA, persistence

## Abstract

Footrot causes 70–90% of lameness in sheep in Great Britain. With approximately 5% of 18 million adult sheep lame at any one time, it costs the UK sheep industry £24–84 million per year. The Gram-negative anaerobe *Dichelobacter nodosus* is the causative agent, with disease severity influenced by bacterial load, virulence, and climate. The aim of the current study was to characterize strains of *D. nodosus* isolated by culture of swabs from healthy and diseased feet of 99 ewes kept as a closed flock over a 10-month period and investigate persistence and transmission of strains within feet, sheep, and the flock. Overall 268 isolates were characterized into strains by serogroup, proline–glycine repeat (pgr) status, and multi-locus variable number tandem repeat analysis (MLVA). The culture collection contained 87 unique MLVA profiles and two major MLVA complexes that persisted over time. A subset of 189 isolates tested for the virulence marker aprV2 were all positive. The two MLVA complexes (76 and 114) comprised 62 and 22 MLVA types and 237 and 28 isolates, respectively. Serogroups B, and I, and pgrB were associated with MLVA complex 76, whereas serogroups D and H were associated with MLVA complex 114. We conclude that within-flock *D. nodosus* evolution appeared to be driven by clonal diversification. There was no association (*P* > 0.05) between serogroup, pgr, or MLVA type and disease state of feet. Strains of *D. nodosus* clustered within sheep and were transmitted between ewes over time. *D. nodosus* was isolated at more than one time point from 21 feet, including 5 feet where the same strain was isolated on two occasions at an interval of 1–33 weeks. Collectively, our results indicate that *D. nodosus* strains persisted in the flock, spread between sheep, and possibly persisted on feet over time.

## Introduction

In England, approximately 5% sheep are lame at any one time. Footrot, caused by *Dichelobacter nodosus* (1), causes 70–90% of lameness and costs the UK sheep industry £24–84 million per year (2, 3). There are two distinct clinical presentations of footrot, an inflammation of the interdigital skin [interdigital dermatitis (ID)] and separation of the hoof horn from the underlying tissue (severe footrot).

A number of approaches have been used to characterize *D. nodosus* strains, isolates, and populations. Historically these have included visual assessment by microscopy and descriptions of colony morphology and growth patterns (4–6). More recently, serotyping, virulence determination, analysis of single and multiple loci, and whole-genome sequencing have been used (7–15).

Several strain types of *D. nodosus* have been detected within and between sheep, flocks (7, 9, 13, 14, 16–18), and geographic regions (9, 13, 14). It has also been reported that clonal groups persist within farms and regions for decades (9); however, there is little information on the persistence of individual strains within sheep or feet. Serogroup E isolates have been detected at up to a 60-day interval from the same sheep by culture (19), and challenge study isolates have been re-isolated from sheep 1 month after challenge (20). Sheep-level persistence beyond this time-scale has not been reported.

Various putative virulence markers can be used in strain typing and have been used to characterize the pathogenicity of isolates of *D. nodosus*. Historically, this has included the phenotypic gelatin-gel and elastase tests (12), and more recently, DNA-based approaches including determination of pgr type or whether an isolate encodes for a “virulent” or “benign” protease variant (aprV2 and aprB2, respectively) (11, 15). However, virulence markers are not all present in all isolates (12), meaning that the same isolate could be classified as “virulent” or “non-virulent” (benign) depending on the test used.

To date, there have been no detailed analyses of a population of *D. nodosus* isolates from healthy and diseased feet of sheep in a single flock over time, this limits understanding of *D. nodosus* population dynamics and associations with footrot within a flock. The aims of the current study were to characterize *D. nodosus* isolates cultured from a flock of 99 sheep over a 10-month period to investigate persistence of strains of *D. nodosus* in feet, sheep and the flock, and associations between strains and disease state of feet.

## Materials and Methods

### Trial Design

The study flock and design are described elsewhere (21). In brief, a flock of 99 crossbred ewes were monitored for 10 months from October 2010 to August 2011. All four feet of every sheep in the flock were examined and the interdigital skin of each foot was swab-sampled at the start and end of the study. At this examination, all feet were scored for footrot (22) and a swab sample was taken. The locomotion of all 99 ewes and their lambs (when present) was scored (23) each week. Throughout the 10-month study, when a sheep was lame [locomotion score (LS) >2 (23)], the feet were scored and a swab sample taken from the interdigital skin and any active footrot lesions. All sheep were treated with either parenteral and topical antibiotic or foot trimmed and topical antibiotic applied, within a week of becoming lame with LS >2. Treated individuals were examined and swab-sampled each week for 2 weeks after treatment.

### Isolation of *D. nodosus*

Samples for culture were collected on sterile wooden sticks and stored in Amies transport medium with charcoal at room temperature and cultured within 2 days (5, 24). Swabs were streaked across 4% hoof agar (HA) and incubated at 37°C in anaerobic jars with an Anaerogen pack and an indicator (AN0025, Thermo Fisher Scientific, Hampshire, UK) for 4–5 days to isolate *D. nodosus*. Up to six individual colonies were selected from each positive culture, inoculated onto 2% HA, and incubated as above for 3 days. Each isolate, with uniform colony morphology and heavy growth, was re-inoculated onto 2% HA and incubated as above. Cultures from these plates were harvested and DNA extracted using the Nucleospin Blood Kit (Macherey-Nagel, Düren, Germany) following the manufacturer’s instructions.

### Molecular Analyses

Isolates were confirmed as *D. nodosus* by PCR using the primers described by La Fontaine et al. (25) (Table S1 in Supplementary Material). For isolates confirmed as *D. nodosus*, pgr status was determined by analysis with the pgrA and pgrB primer sets (Table S1 in Supplementary Material) (11); and serogroups were determined using a multiplex PCR approach (Table S1 in Supplementary Material) (26). All *D. nodosus* isolates were genotyped using a multi-locus variable number tandem repeat analysis (MLVA) typing scheme (14). As in Russell et al. (14), the number of repeats in the DNTR 09, 10, and 19 regions were determined by agarose gel electrophoresis and for DNTR02 by sequencing using the Tandem Repeats Finder at https://tandem.bu.edu/trf/trf.html [last accessed March 10, 2016 (27)]. The presence of the aprV2/B2 virulence marker in 189 isolates was determined by amplification and sequencing of a fragment to identify the dinucleotide polymorphism that discriminates between aprV2 and aprB2 genes (Table S1 in Supplementary Material) (28). Representative sequences of each detected DNTR02 allele have been submitted to Genbank under Accession numbers KU892102–KU892125.

### Data Recording and Analysis

The disease state of each foot was classified as healthy (ID score = 0), mild ID (ID score 1 or 2), severe ID (ID score 3 or 4), or footrot (footrot score ≥1 anywhere on the foot irrespective of ID score) (22). The number of isolates and rate of isolation were calculated. Each isolate was characterized to a strain by serogroup, pgr, and MLVA type.

The discriminatory ability (*D*) of serogroups, pgr, and MLVA loci and types was calculated as described by Hunter and Gaston (29), e.g., if *D* = 0.7, there was a 70% probability that two isolates selected at random would belong to different types. The culture collection was analyzed as a single population, divided into MLVA complexes (MC), using the global optimal eBURST (goeBURST) algorithm (30). This was visualized as a minimum-spanning tree (MST) in PHYLOViZ (31). The MST was supplemented with isolate metadata, including serogroup, pgr, and disease state. MLVA complexes were named on the basis of the predicted founder strain. Where there was no predicted founder strain they were classed as minor groups and numbered arbitrarily. Within MLVA complexes, subgroup founder strains, defined as those that have ≥3 single locus variants were also identified. MLVA types represented by more than five isolates were used to investigate the distribution of MLVA types overall, by lesion score, serogroup, and pgr variant. Only one MLVA complex (MC76; *n* = 237) contained strains that were represented by more than five isolates, so all isolates from a second MLVA complex (MC114; *n* = 28) were included for comparison.

The distributions of serogroup, pgr, and MLVA type by disease state were investigated using Chi-squared, Fisher’s Exact Tests, and Monte Carlo estimations of exact values in SPSS (IBM SPSS Statistics, 64-bit edition, release 22.0.0.0). Where significance was reached, categories were tested individually and the Bonferroni correction applied to correct for multiple testing (IBM SPSS Statistics, 64-bit edition, release 22.0.0.0). *D. nodosus* were classified into strain types based on serogroup, pgr, and MLVA type for each isolate. This strain type was used to investigate the persistence and distribution of strains on feet and sheep at one time point and over time.

## Results

A total of 4,801 swab samples were collected for bacteriological examination; 4,012 from interdigital skin and 789 from footrot lesions. From these, 390 fully typeable (serogroup, pgr, and MLVA type) cultures were isolated from 195 (4.1%) swabs. Five isolates produced ambiguous pgr results and four produced ambiguous serogroup results and were excluded from further analysis. There were 113 isolates that were duplicates (isolated from the same swab with identical serogroup, pgr, and MLVA type) and were removed from the analysis. This resulted in a final dataset of 268 isolates from 157 interdigital skin and 35 footrot lesion swabs; this included one occasion where the same strain was isolated from a footrot lesion and the interdigital skin of the same foot at the same time.

### Serogroup

Four serogroups were detected during the trial: B, D, H, and I. Serogroup B was present in 226/268 (84.3%) isolates (Table 1). Serogroups B, H, and I were detected throughout the study, whereas serogroup D was first isolated in January 2011. There was no significant difference in the distribution of serogroup type by disease state of feet (Table 1; Monte Carlo estimate *P* = 0.51). Serogroup diversity (*D*) was 0.28, indicating that if two isolates were selected at random from the population, on 28% of occasions they would belong to different serogroups. There were 59 swabs with more than one strain isolated, and 19 (32.2%) of these included more than one serogroup, which was not significantly different from expectation (Fisher’s Exact Test *P* = 0.69).

**Table 1:** **Serogroup and pgr distribution of 268 isolates by disease state**.

Disease state	*n* (%)	Serogroup				pgr	
		B	D	H	I	A	B
Healthy	64 (23.9)	52	1	8	3	43	21
Mild ID	56 (20.9)	50	1	2	3	31	25
Severe ID	53 (19.8)	48	0	3	2	35	18
Footrot^a^	48 (17.9)	36	1	5	6	31	17
Lesion^b^	47 (17.5)	40	0	5	2	28	19
Total	268	226	3	23	16	168	100

*ID, interdigital dermatitis*.

*^a^Interdigital skin sample*.

*^b^Non-interdigital sample*.

### Putative Virulence Markers

pgrA and pgrB variants were detected throughout the study; pgrA was present in 168/268 (62.7%) isolates and pgrB in 100/268 (37.3%) isolates (Table 1). There was no significant difference in the distribution of pgr variants by disease state (Monte Carlo estimate *P* = 0.68). There was a 47% chance of selecting different pgr types at random from the population. There were 70 swabs with pgrA only and 63 with pgrB only. Of the 59 swabs with >1 strain, 32 contained pgrA only, 6 contained pgrB only, and 21 (35.6%) contained both pgrA and pgrB. This distribution differed significantly from expectation (Monte Carlo estimate *P* < 0.01), indicating that pgrA and pgrB were not randomly distributed and fewer swabs had only pgrB.

The 189 isolates tested for aprV2/aprB2 included 168 isolates present in the final dataset. This was 62.7% of the final isolate dataset and included examples of all four serogroups, both pgr types, and 79/87 (90.8%) of MLVA types. All isolates contained aprV2.

### Multi-Locus Variable Number Tandem Repeat Analysis

The 268 isolates comprised 87 distinct MLVA types (Table S2 in Supplementary Material), and the discriminatory ability (*D*) of the four loci ranged from 0.56 to 0.82, and was 0.89 overall (Table S3 in Supplementary Material). There was an overdispersed Poisson distribution of isolates by MLVA type (mean = 3.1, variance = 80.3) with one MLVA type (62) representing 30.6% (82/268) of the population; and the seven most frequently detected MLVA types (*n* > 5) represented 54.1% (145/268) of the population (Table S4 in Supplementary Material). Of the 59 swabs with more than one strain, 56 (95%) contained more than one MLVA type, not different from expectation (Fisher’s Exact Test *P* = 0.49).

**Figure 1:**
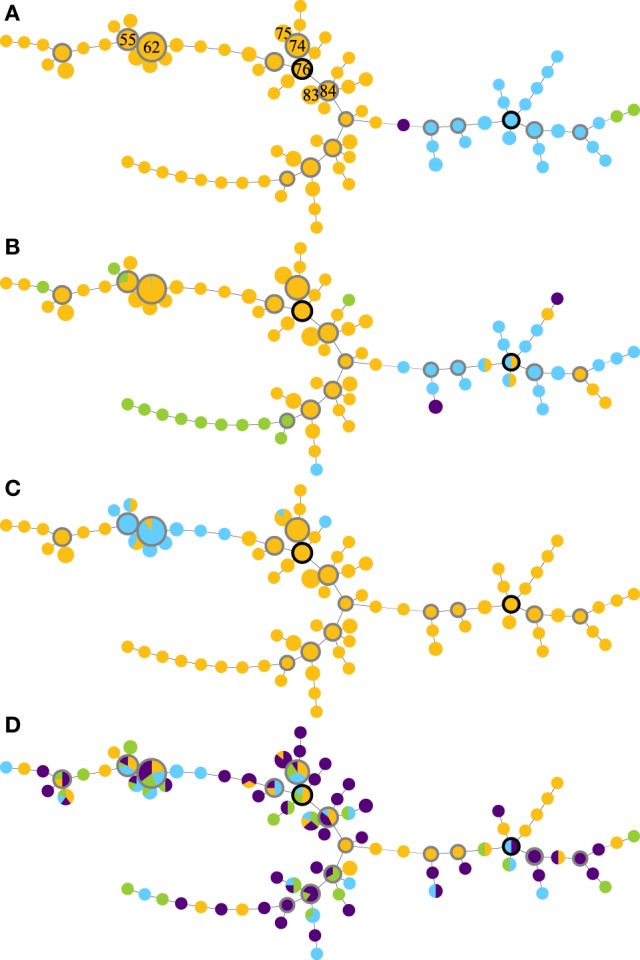
**Minimum-spanning trees of MLVA profiles (A), supplemented with data on serogroups (B), pgr status (C), and disease state (D) of *Dichelobacter nodosus* cultures isolated from a flock of 99 ewes repeatedly sampled over 10 months**. The seven most frequently isolated MLVA types are indicated by number in panel **(A)**; predicted founder strains have a black border and subgroup founder strains a gray border, and circle size is proportional to numbers of isolates. Colors indicate **(A)** MLVA complexes: MC76 (orange); MC114 (blue); minor group I (green); and singleton (purple). Lines between MLVA profiles in the same MLVA complex indicate single locus variants, and between isolates in different MLVA complexes indicate double locus variants. **(B)** Serogroup: B (orange), H (blue), I (green), and D (purple). **(C)** pgr status: A (orange) and B (blue). **(D)** Foot disease state: healthy (orange), mild interdigital dermatitis (ID) (blue), severe ID (green), and severe footrot including lesions (purple).

Global optimal eBURST analysis of the MLVA profiles revealed two large MLVA complexes (MC), one minor group and one singleton (Figure 1A). MLVA complex 76 contained 237/268 isolates and 62/87 MLVA types, including the seven most frequently detected MLVA types. MLVA complex 114 contained 28/268 isolates and 22/87 MLVA types; both MLVA complexes contained a number of subgroup founders. The minor group contained two MLVA types and two isolates, and the singleton was one MLVA type represented by a single isolate (Figure 1A).

The distribution of serogroups within MLVA complexes differed significantly (Monte Carlo estimate *P* < 0.01), with isolates in MC76 most likely to be Serogroup B or I, and isolates in MC114 most likely to be Serogroup D or H (Figure 1B). The distribution of pgr variants by MLVA complex also differed from expectation (Fisher’s Exact Test, *P* < 0.01). pgrB variants were only detected within MC76, and 98.0% (98/100) of these variants clustered around subgroup founder MLVA types 62 and 55 and represented 86.7% (98/113) of the isolates around these subgroup founders (Figure 1C). There was no association between the MLVA type of strains isolated from interdigital skin by disease state (Monte Carlo estimate *P* = 0.51; Figure 1D).

### Multiple Isolates at a Single Time point

There were 96 strain types (serogroup, pgr, and MLVA type), with *D* = 0.91. The number of strains isolated from swabs followed a Poisson distribution, with one strain isolated from 133 swabs, two from 46 swabs, three from 10 swabs, four from two swabs, and one swab that yielded five strains. There was no significant association between the number of strains isolated from a swab and foot disease state (Monte Carlo estimate *P* = 0.48).

In 30 ewes and 8 lambs, *D. nodosus* was isolated from more than one foot at the same time point. There were up to eight strains isolated per individual, and on 14/38 occasions the same strain was detected on more than one foot, significantly more often than expected (Fisher’s Exact Test *P* < 0.01). On 9/14 occasions, the dominant BB62 strain (serogroup B, pgrB, MLVA type 62) was among the strains identified, this was not significantly different from expectation (Fisher’s Exact Test *P* > 0.05).

### Repeat Isolations from Sheep and Feet over Time

Over the trial period, *D. nodosus* was isolated from 67 ewes and 21 lambs; isolates were cultured on one occasion from 34 ewes and all 21 lambs, on two occasions from 23 ewes, on three occasions from 8 ewes and on four occasions from 2 ewes (Table 2; Table S5 in Supplementary Material). There were, therefore, 45 animal-level repeat isolation events, 21/45 of these were from the same foot at different time points (Table 2). On 16/45 occasions (35.6%), the same strain was isolated from an animal more often than expected (Fisher’s Exact Test *P* < 0.01); and on 5/16 occasions this was from the same foot. For 13/16 repeat strain isolation events (81.3%), including four on the same foot, the population dominant strain (BB62) was isolated (Table 3). The inter-isolation period for BB62 ranged from 1 to 36 weeks for the same animal and from 2 to 33 weeks from the same foot. Not all ewes were treated for footrot between isolation events, those that were, were treated up to three times.

**Table 2:** **Frequency of isolation of any *Dichelobacter nodosus* from an animal and foot, and by strain with the inter-isolation interval and number of treatments for footrot when there was >1 isolation event**.

		Animal		Same foot (same strain)
		Ewe	Lamb	
Number of isolation events	1	34	21	
	2	23	0	19 (5)
	3	8	0	1
	4	2	0	
Inter-isolation interval (weeks)	Min	1		1 (1)
	Max	42		42 (33)
	Mode	1		20 (nc^a^)
Number of treatments for footrot	Min	0		1 (1)
	Max	5		5 (3)
	Mode	1		1 (1)

*^a^nc: not calculable, all five values were different (1, 2, 14, 20, 33)*.

**Table 3:** **Repeat isolation by strain type BB62 (serogroup B, pgrB, MLVA type 62) and other strain types**.

		First isolation event		Total
		BB62	Non-BB62	
Second isolation event	BB62	13	3	16
	Non-BB62	7	22	29
Total		20	25	45

## Discussion

This is the first study to isolate and strain type *D. nodosus* and examine spread and persistence of strains within feet, sheep, and a flock over time. Strain typing using a combination of serogroup, pgr, and MLVA type gave an objective code to track individual strains in feet and within and between sheep, over time. Key results include clonal expansion of *D. nodosus* within the flock, clustering of strains within sheep at a single time point, spread of *D. nodosus* between feet over time, and possible persistence of *D. nodosus* strains on feet over time. There were no strains statistically associated with any disease state.

The coexistence of two MLVA complexes throughout the study indicate that strains were relatively stable at flock level over the 10-month trial period, supporting Buller et al. (9). Diversity within the strain collection and the non-random clustering of serogroups and pgrB (Figures 1B,C) indicate that within-flock evolution of *D. nodosus* was driven by clonal expansion as reported elsewhere (9, 14, 32). The limited diversity of *D. nodosus* strains within the flock means that evolution by recombination (13, 14), is plausible, however, the maintenance and dominance of two distinct MLVA complexes suggests that recombination did not play a major role in *D. nodosus* diversification in this flock.

There was no association between any strain typing approach (serogroup, pgr, MLVA, or aprV2) and disease state in the current study. The aim of the MLVA scheme is to describe population diversity and neutral variation and avoid use of markers in virulence-associated regions (14). Serogroups *per se* are not associated with disease state, although a serogroup is sometimes correlated with disease in a closed population (32). All isolates tested in the current study were positive for the aprV2 protease virulence marker as is common in GB (7) and indicate that aprV2 is not a useful marker for severe footrot in GB, unlike the situation in Norway where one aprV2-positive serogroup was associated with their footrot epidemic (32). There was an association between pgrA and disease in a global study of *D. nodosus* (11), but not in the current study. This might be because of competitive interaction between strains [e.g., reviewed in Ref. (33)] that, in a closed flock, led to the clustering of pgr alleles at the foot-level. Alternatively, this could be an ecological fallacy (34), i.e., what occurs at a large spatial scale could differ from what occurs on an individual foot, and global studies might not infer correctly to individuals. This parallels the global finding of recombination driving *D. nodosus* evolution, but clonal diversification occurring within flocks (14). Consequently, the current study of a small number of sheep in a closed population with frequent disease has not elucidated strain variation associated with disease state. This could be a real effect; in which case, the factors “causing” disease were not those we tested. Equally, this could be due to low statistical power because of the low rate of isolation of *D. nodosus* or be because we focused a detailed study on a single flock (for a limited time period) and a greater number of study flocks (or a longer time period) might have revealed further insights.

Individual strains persisted within the flock throughout the trial, and the majority of identical isolates were detected on different ewes over time indicating that strains were spreading between sheep. This supports the theory of frequent transmission and infection/colonization of feet. There was some evidence to suggest that strains might persist on a foot, although the time interval between re-isolation of the same strain from the same foot was highly variable. The longest interval (with no intermediate isolation events) was 33 weeks, longer than the 1- to 2-month persistence reported elsewhere (19, 20). However, it is possible that this was due to re-colonization/re-infection by the same strain rather than persistence.

## Conclusion

A total of 96 strains of *D. nodosus* were isolated from 192 *D. nodosus*-positive swabs in a 10-month study of a single flock of 99 ewes. Diversification of *D. nodosus* in this flock appeared to be driven by clonal expansion, two MLVA complexes coexisted within the flock and a small number of strains dominated the population of *D. nodosus*. Several different strains were present on individual feet of the diseased and healthy, and no one strain was statistically associated with disease state. Strains of *D. nodosus* persisted throughout the study and spread between feet; there was inconclusive evidence that they persisted on individual feet over time.

## Ethics Statement

This study was carried out in accordance with the recommendations for “Ethical Approval of an Investigation Involving Animals,” University of Bristol Ethics Committee. The protocol was approved by the University of Bristol Ethics Committee, UIN number UB/09/020.

## Author Contributions

LG and RG-T designed the study with input from GM. ES managed the fieldwork with assistance from CR. CR, ES, and MM conducted the laboratory work; ES and AG conducted the statistical analysis and drafted the manuscript. LG and KP contributed to the interpretation of the data. All authors contributed to the manuscript editing and approved the final manuscript.

## Conflict of Interest Statement

The authors declare that the current research was conducted in the absence of any commercial or financial relationships that could be construed as a potential conflict of interest.
