# Supplementary material for: Within-Flock Population Dynamics of Dichelobacter nodosus
Source: Front Vet Sci. 2017 Apr 24;4:58. doi: 10.3389/fvets.2017.00058 (PMC5401886; doi:10.3389/fvets.2017.00058)
Supplement: Supplementary file 1 [file Table_1.pdf]

Supplementary Material

Within-flock population dynamics of *Dichelobacter nodosus*

Edward M. Smith, Andrew Gilbert, Claire L. Russell, Kevin J. Purdy, Graham F. Medley, Mohd Muzafar, Rose Grogono-Thomas and Laura E. Green[*]

*** Correspondence:**
Laura E. Green
Laura.Green@warwick.ac.uk

Supplementary table 1. Sequences and source of primers used for PCR and sequencing

Target	Primer	Sequence (5' – 3')	Product size (bp)	Reference
D. nodosus	Ac<br>Cc	TCGGTACCGAGTATTTCTACCCAACACCT<br>CGGGGTTATGTAGCTTGC	783	La Fontaine et al 1993
pgrA	pgrA_F<br>pgrA_R	GAAGCTTTCGAGAGA<br>CAACAACTGCATCAGCA	Variable	Calvo-Bado et al 2011
pgrB	pgrB_F<br>pgrB_R	AMCATCYGGAAAGGTGA<br>GACGGCATCAGCAGCA	Variable	
aprV2 / aprB2	Dnod_Apr-L<br>Dnod_Apr-R	TCCAACCGCTGCTCCAAATG<br>ACCACCGCAACGACCCAATG	436	Stäuble et al 2014
Serogroups	Universal F	CCTTAATCGAACTCATGATTG		Dhungyel et al 2002
Serogroup A	SeroARev	AGTTTCGCCTTCATTATATTT	415	
Serogroup B	SeroBRev	CGGATCGCCAGCTTCTGTCTT	283	
Serogroup C	SeroCRev	AGAAGTGCCTTTGCCGTATTC	325	
Serogroup D	SeroDRev	TGCAACAATATTTCCCTCATC	319	
Serogroup E	SeroERev	CACTTTGGTATCGATCAACTTGG	363	
Serogroup F	SeroFRev	ACTGATTTCGGCTAGACC	241	
Serogroup G	SeroGRev	CTTAGGGGTAAGTCCTGCAAG	279	
Serogroup H	SeroHRev	TGAGCAAGACCAAGTAGC	409	
Serogroup I	SeroIRev	CGATGGGTCAGCATCTGGACC	189	
DNTR02	DNTR02F<br>DNTR02R	GATCCATCGTTTCATCGTCA<br>CGCACTTTAGCCGTTATGTTT	Variable	Russell et al 2014
DNTR09	DNTR09F<br>DNTR09R	GGCGTAAACGAAATGCCTAA<br>ATCGGCGGAAGATTGTCTC	Variable	
DNTR10	DNTR10F<br>DNTR10R	CCGTCTATCCACCCGATTTA<br>TTGAACCGCGTCACTATCAG	Variable	
DNTR19	DNTR19F<br>DNTR19R	CCCGTCGAATCACTCCAG<br>GGTAGCGCCGAAGAAAGA	Variable	
