# Supplementary material for: Within-Flock Population Dynamics of Dichelobacter nodosus
Source: Front Vet Sci. 2017 Apr 24;4:58. doi: 10.3389/fvets.2017.00058 (PMC5401886; doi:10.3389/fvets.2017.00058)
Supplement: Supplementary file 2 [file Table_2.pdf]

Supplementary Material

Within-flock population dynamics of *Dichelobacter nodosus*

Edward M. Smith, Andrew Gilbert, Claire L. Russell, Kevin J. Purdy, Graham F. Medley, Mohd Muzafar, Rose Grogono-Thomas and Laura E. Green*

*** Correspondence:**
Laura E. Green
Laura.Green@warwick.ac.uk

Supplementary table S2. *D. nodosus* MLVA allelic profiles

	Number of tandem repeats			
**MLVAType**	**DNTR02**	**DNTR09**	**DNTR10**	**DNTR19**
49	6	6	10	3
50	6	6	11	3
51	6	6	11	4
52	6	6	12	3
53	6	7	11	4
54	6	7	12	3
55	6	7	12	4
56	6	7	12	5
57	7	6	11	3
58	7	7	10	3
59	7	7	11	3
60	7	7	11	4
61	7	7	12	3
62	7	7	12	4
63	7	7	12	5
64	8	7	12	4
65	9	5	4	4
66	9	5	6	5
67	9	7	12	4
68	10	5	11	3
69	10	6	10	3
70	10	6	10	4
71	10	6	12	3
72	11	5	4	4
73	11	5	11	3
74	11	6	10	3
75	11	6	10	4
76	11	6	11	3
77	11	6	11	4
78	11	6	12	3
79	11	6	12	4
80	11	7	11	4
81	11	7	12	4
82	12	5	11	3
83	12	6	10	3
84	12	6	11	3

Table S2 (*cont*). *D. nodosus* MLVA allelic profiles

	Number of tandem repeats			
**MLVAType**	**DNTR02**	**DNTR09**	**DNTR10**	**DNTR19**
85	12	6	11	4
86	12	6	12	3
87	12	6	12	4
88	13	5	4	4
89	13	6	6	4
90	13	6	10	3
91	13	6	11	3
92	13	6	12	3
93	14	5	6	4
94	14	5	6	5
95	14	5	11	3
96	14	6	10	3
97	14	6	11	3
98	14	6	11	5
99	14	6	12	3
100	14	6	12	4
101	15	5	12	5
102	15	6	10	3
103	15	6	10	5
104	15	6	11	3
105	15	6	12	3
106	15	6	12	5
107	16	5	6	4
108	16	5	6	5
109	17	5	6	4
110	18	4	5	5
111	18	5	4	4
112	18	5	5	5
113	18	5	6	3
114	18	5	6	4
115	18	5	6	5
116	19	5	5	4
117	19	5	5	5
118	19	5	6	5
119	19	6	9	4
120	20	5	5	5
121	20	5	6	4
122	21	5	5	5
123	21	6	9	4
125	22	6	6	3
126	22	6	11	3
127	24	6	11	3
128	25	5	6	4
129	28	6	10	3
130	28	6	12	3
131	31	6	11	3
132	36	6	12	3
133	39	6	10	3
134	39	6	11	4
135	40	6	10	3
136	40	6	11	3
