# Supplementary material for: Within-Flock Population Dynamics of Dichelobacter nodosus
Source: Front Vet Sci. 2017 Apr 24;4:58. doi: 10.3389/fvets.2017.00058 (PMC5401886; doi:10.3389/fvets.2017.00058)
Supplement: Supplementary file 3 [file Table_3.pdf]

Supplementary Material

Within-flock population dynamics of *Dichelobacter nodosus*

Edward M. Smith, Andrew Gilbert, Claire L. Russell, Kevin J. Purdy, Graham F. Medley, Mohd Muzafar, Rose Grogono-Thomas and Laura E. Green*

*** Correspondence:**
Laura E. Green
Laura.Green@warwick.ac.uk

Supplementary table S3. Discriminatory ability [*D*] of each MLVA locus and overall

	Discriminatory ability (*D*)	No. alleles / types
MLVAType	0.895	87
DNTR02	0.821	24
DNTR09	0.606	4
DNTR10	0.688	7
DNTR19	0.565	3
