# Supplementary material for: Within-Flock Population Dynamics of Dichelobacter nodosus
Source: Front Vet Sci. 2017 Apr 24;4:58. doi: 10.3389/fvets.2017.00058 (PMC5401886; doi:10.3389/fvets.2017.00058)
Supplement: Supplementary file 4 [file Table_4.pdf]

Supplementary Material

Within-flock population dynamics of *Dichelobacter nodosus*

Edward M. Smith, Andrew Gilbert, Claire L. Russell, Kevin J. Purdy, Graham F. Medley, Mohd Muzafar, Rose Grogono-Thomas and Laura E. Green*

*** Correspondence:**
Laura E. Green
Laura.Green@warwick.ac.uk

Supplementary table S4. Frequency of MLVA types detected in 10-month study of a population of 99 ewes

Frequency of isolates	No. of MLVA types	MLVA code
1	57	51, 52, 54, 56, 57, 58, 59, 65, 67, 68, 69, 70, 71, 72, 77, 80, 81, 82, 85, 88, 89, 90, 91, 92, 93, 94, 95, 98, 99, 100, 101, 103, 106, 107, 108, 110, 111, 112, 116, 117, 119, 120, 121, 122, 123, 125, 126, 127, 128, 129, 130, 131, 132, 133, 134, 135, 136
2	12	53, 61, 64, 66, 73, 86, 87, 109, 113, 114, 115, 118
3	5	63, 79, 96, 97, 105
4	3	50, 78, 102
5	3	49, 60, 104
6	1	76
7	2	75, 84
11	2	55, 83
21	1	74
82	1	62
