# Supplementary material for: Within-Flock Population Dynamics of Dichelobacter nodosus
Source: Front Vet Sci. 2017 Apr 24;4:58. doi: 10.3389/fvets.2017.00058 (PMC5401886; doi:10.3389/fvets.2017.00058)
Supplement: Supplementary file 5 [file Table_5.pdf]

Supplementary Material

Within-flock population dynamics of *Dichelobacter nodosus*

Edward M. Smith, Andrew Gilbert, Claire L. Russell, Kevin J. Purdy, Graham F. Medley, Mohd Muzafar, Rose Grogono-Thomas and Laura E. Green*

*** Correspondence:**
Laura E. Green
Laura.Green@warwick.ac.uk

Supplementary table S5. Strains of *D. nodosus* isolated from the same sheep over time

Sheep	First isolation	Interval to next isolate (wks)	Number of episodes of footrot	Second isolation	Interval to next isolate (wks)	Number of episodes of footrot	Third isolation	Interval to next isolate (wks)	Number of episodes of footrot	Fourth isolation
066	**BB62→**	2	1	**←BB62**						
117	BA50; BA51; BB64; **BB63→**	21	2	BA102; BA105; **←BB63**; BB65						
119	**BB62→** HA108	20	1	**←BB62**						
121	BB61; **BB62→** BA99; BA103; BA106; BA122	14	1	**←BB62**						
124	BA76; BA84; **BB62→**	36	1	BA62; **←BB62**						
137	**BB62→**	1	1	**←BB62**						
142	**BB62→** BA104; HA114	33	3	**←BB62**						
147	BA84; **BB62→** BA97; BA104; HA128; HB62; IA57; IA136	36	3	**←BB62**; BA114; HA110						
076	**BA83→** HA121	1	0	**←BA83**	41	4	BA49; BA52; BA74			
113	HA113	2	1	**BB62→**	9	3	**←BB62**			

Sheep	First isolation	Interval to next isolate (wks)	Number of episodes of footrot	Second isolation	Interval to next isolate (wks)	Number of episodes of footrot	Third isolation	Interval to next isolate (wks)	Number of episodes of footrot	Fourth isolation
106	BA75; IA135	19	0	**BA50→**	1	1	**←BA50**			
134	HA123	19	1	**BB62→**	23	0	**←BB62** BA74; BA76; BA78; BB60			
135	HA119	28	0	**BB62→**	14	1	**←BB62**; BA62			
111	BB61; **BA102→**	12	3	BA104; DA66	29	4	BA49; BA74; BA105; BA113; **←BA102;** BB64			
069	BA84	8	2	**BB62→**	2	0	**←BB62→**	18	1	**←BB62**
140	BA74; HA109	19	2	**BB62→**	11	1	**←BB62**; BA68	12	1	BA78
059	BA83	42	2	BB53; IA132; BA53; BB55						
065	BB62	35	3	IB55						
071	IA134	25	1	BA54						
081	HA115	32	2	BA60; BA62; BA74						
089	BB62	30	1	BA74; IB56						
092	BB62	30	1	BA60; BA62						
094	BB62	1	1	BA84						
103	BB62	28	3	BB55						
104	BB67; HA101	7	5	IA131						
107	BA96; HA89; HA93	42	2	BA74						
108	IA127	42	0	BA74						
112	BA74	13	3	BB63						
114	HA94; HA118	6	1	BB62						
143	BA62; BA79	33	1	BB62						
149	BB60	37	2	BA74						
067	HA118	8	1	BB62	23	1	BA62; BB55; BA76; BA109			
077	BB62	6	1	BA62	20	4	BA72; BA74; BA96; BB55; HA88; IA130			

Isolates are named based on serogroup, pgr status and MLVA type; e.g. BA104 is serogroup B, pgrA, MLVA type 104. Strain names in bold and arrows indicate putative incidences of persistence.
